# How to manage work-related stress in healthcare professionals: organizational and individual interventions.

**DOI:** 10.1192/j.eurpsy.2024.1250

**Published:** 2024-08-27

**Authors:** P. Catapano, M. Luciano, S. Cipolla, S. Pascolo, G. Sampogna, F. Perris, F. Catapano, A. Fiorillo

**Affiliations:** Department of Psychiatry, University of Campania “Luigi Vanvitelli”, Naples, Italy

## Abstract

**Introduction:**

Workplaces can be source of significant stress for employees, leading to a series of mental health problems, such as burnout syndrome. Healthcare professionals and other helping professions are especially vulnerable to work-related stress.

**Objectives:**

The aim of the present review is to assess available intervention aiming at improving work-related stress symptoms.

**Methods:**

We conducted a thorough search of relevant articles on PubMed, APA PsycInfo, and Scopus databases, using specific keywords such as “occupational stress,” “stress,” “anxiety,” “depression,” “health personnel,” “health care facilities, manpower and services,” “prevention,” and “control.”

**Results:**

Although significant methodological heterogeneity has been found among studies, regarding assessment tools, target population, and intervention types, we can still draw some satisfactory results. Healthcare professionals have access to various interventions to manage work-related stress symptoms, which can be classified into three categories: 1) individual cognitive-behavioral therapy approaches, 2) relaxation techniques at the individual level, and 3) organizational-level interventions. Mindfulness techniques, relaxation techniques, emotional freedom techniques, and integrated interventions have demonstrated effectiveness in alleviating work-related stress.

**Conclusions:**

To prevent work-related stress among healthcare professionals, interventions should be targeted towards specific categories of healthcare workers based on factors such as age, tasks, and patient types. Well-structured and reliable randomized controlled trials focusing on the most promising interventions, such as mindfulness, need to be carried out in larger samples and with a solid methodology in order to confirm these evidences.

**Disclosure of Interest:**

None Declared

